# ARF6 Promotes the Formation of Rac1 and WAVE-Dependent Ventral F-Actin Rosettes in Breast Cancer Cells in Response to Epidermal Growth Factor

**DOI:** 10.1371/journal.pone.0121747

**Published:** 2015-03-23

**Authors:** Valentina Marchesin, Guillaume Montagnac, Philippe Chavrier

**Affiliations:** 1 Institut Curie, Research Center, 75005 Paris, France; 2 Membrane and Cytoskeleton Dynamics, Centre National de la Recherche Scientifique UMR144, 75005 Paris, France; West Virginia University, UNITED STATES

## Abstract

Coordination between actin cytoskeleton assembly and localized polarization of intracellular trafficking routes is crucial for cancer cell migration. ARF6 has been implicated in the endocytic recycling of surface receptors and membrane components and in actin cytoskeleton remodeling. Here we show that overexpression of an ARF6 fast-cycling mutant in MDA-MB-231 breast cancer-derived cells to mimick ARF6 hyperactivation observed in invasive breast tumors induced a striking rearrangement of the actin cytoskeleton at the ventral cell surface. This phenotype consisted in the formation of dynamic actin-based podosome rosette-like structures expanding outward as wave positive for F-actin and actin cytoskeleton regulatory components including cortactin, Arp2/3 and SCAR/WAVE complexes and upstream Rac1 regulator. Ventral rosette-like structures were similarly induced in MDA-MB-231 cells in response to epidermal growth factor (EGF) stimulation and to Rac1 hyperactivation. In addition, interference with ARF6 expression attenuated activation and plasma membrane targeting of Rac1 in response to EGF treatment. Our data suggest a role for ARF6 in linking EGF-receptor signaling to Rac1 recruitment and activation at the plasma membrane to promote breast cancer cell directed migration.

## Introduction

Reorganization of the actin cytoskeleton is critical for invasive cell behavior of metastatic cancer cells by generating forces needed to move and to overcome physical resistance of three-dimensional (3D) tissue environment [1, 2]. Cancer cells plated on 2D matrix substrates *in vitro* form typical plasma membrane protrusive structures including lamellipodia, sheet-like protruding leading edge of migrating cells and invadopodia, ventral degradative protrusions enriched in membrane-type 1 matrix metalloproteinase (MT1-MMPs), the enzyme responsible for pericellular degradation of ECM components [3]. Lamellipodia and invadopodia are dependent on the assembly of a branched filamentous (F-)actin network and requires activation of the Arp2/3 complex by its upstream activators SCAR/WAVE complex and N-WASP, respectively [4–7]. In addition, growth factors stimulation triggers the formation of highly dynamic actin-based circular dorsal ruffles (CDRs), which propagate as waves along the dorsal plasma membrane. Although their function remains ill-defined, these structures are thought to have a role in fast disassembly and remodeling F-actin prior lamellipodium formation and therefore they may be involved in directed cell motility and internalization of receptor tyrosine kinases (RTKs) and other membrane receptors [8, 9].

The protrusive and migratory potential of invasive tumor cells is enhanced by up-regulation of several genes involved in cell motility including the gene coding for the F-actin binding protein cortactin [10, 11]. In addition, some RTKs such as the epidermal growth factor receptor (EGF-R) have a pro-migratory potential; EGF-R is expressed in carcinoma cells of various origins and it stimulates actin-driven membrane protrusions in response to EGF during chemotactic cell migration [12–14]. EGF-R signaling has been correlated with invasiveness and poor survival in breast cancers [15].

The small GTP-binding protein ADP-ribosylation factor 6 (ARF6) is known to coordinate endocytosis, post-endocytic recycling and actin cytoskeletal organization at the plasma membrane [16, 17]. By controlling recycling of lipids and proteins in the endocytic pathway, ARF6 has been implicated in several cell polarization events including cell migration and cancer invasion. Recent studies suggest a link between up-regulation of ARF6 expression and activity and the invasive capacity of breast cancer cells [18–21]. In human MDA-MB-231 breast adenocarcinoma-derived cells, ARF6 controls invadopodia assembly and the recruitment of invadopodial components cortactin and paxillin through its downstream effector AMAP1 [22]. In addition, ARF6 activation has been linked to EGF signaling via its guanine exchange factor (GEF) GEP100/BRAG2, which interacts with ligand-activated EGF-R [18]. Several studies also showed that ARF6 stimulates actin reorganization and membrane ruffling and promotes the acquisition of a migratory phenotype likely through recycling and activation of the Rho GTPase Rac1 [23–27]. Rac1 promotes actin polymerization during lamellipodia extension by activating the Arp2/3 activator WAVE and by increasing actin monomer availability by regulating cofilin, an F-actin-depolymerization factor [28]. Rac1 is overexpressed or hyperactive in breast tumors [29] and Rac1-GEFs are overexpressed in high-grade poor-prognosis breast tumors [30]. However, ARF6-dependent Rac1 regulation is complex and varies depending on cell types and requires further investigation [27, 31, 32].

In this study we used a gain of function approach to mimic up-regulation of ARF6 activity reported in breast tumor cells and to unravel the mechanism through which ARF6 controls cancer cell migration. In MDA-MB-231 cells, expression of a hyperactive mutant of ARF6 (ARF6T157N) [33, 34] induced a striking rearrangement of the actin cytoskeleton at the ventral cell surface, consisting in the formation of podosome rosette-like structures positive for markers including cortactin, the Arp2/3 complex, the SCAR/WAVE complex and its most prominent regulator Rac1. In addition, we showed that ARF6 silencing inhibits Rac1 activation and targeting to the leading edge in response to EGF treatment, suggesting a role for ARF6 in linking EGF-R signaling to Rac1 recruitment and activation at the plasma membrane.

## Materials and Methods

### Cell culture

Human breast adenocarcinoma MDA-MB-231 cells (American Type Culture Collection HTB-26) were maintained in L-15 culture medium (Sigma–Aldrich, St. Louis, MO, USA) with 2 mM glutamine (GIBCO) and 15% FBS (GIBCO) at 37°C in 1% CO_2_.

### Immunoblotting analysis

Cells were lysed and proteins were eluted in SDS sample buffer, separated by SDS-PAGE, and detected by immunoblotting analysis with indicated antibodies. Bound antibodies were detected with ECL Western Blotting Detection Reagents (GE Healthcare Life Sciences). Densitometric quantification of bands was performed with Gels tool of ImageJ software (http://rsb.info.nih.gov/ij/).

### Antibodies

Antibodies used in this study are listed in S1 Table.

### Plasmid constructs and DNA transfection

Full-length rat cortactin cDNA subcloned in pDsRed1-N1 or pEGFP-N1 (Clonetech) were provided by Dr M.A. McNiven (Mayo Clinic, Rochester, MI, USA). cDNA encoding ARF6T157N (a gift from Dr M. Franco, Univ Sophia-Antipolis, France) was inserted in a pDEST WPI lentiviral expression vector (a kind gift from P. Benaroch, Institut Curie, Paris) through Gateway Cloning technology (Invitrogen). C-terminally GFP-tagged ARF6T157N construct was obtained by subcloning ARF6T157N cDNA into a pEGFP-N1 vector (Clontech). N-terminally-GFP tagged Rac1G12V was a gift from P. Fort (CRBM, Montpellier, France). For transient expression, MDA-MB-231 cells were transfected with plasmid constructs (1 μg) by using Lipofectamine LTX (Invitrogen) or Nucleofector (Lonza) according to the manufacturer’s instructions. Cells were analyzed 48 h after transfection.

### Protein purification and pull-down assay

pGEX constructs encoding GST or the residues 371–507 (LZII domain) of human JIP3 fused with an amino-terminal GST moiety [35] were purified as described [35].

MDA-MB-231 cells or MDA-MB-231 cells stably overexpressing ARF6 wild-type, ARF6T27N or ARF6T157N were lysed in 50 mM Tris pH 7.4, 137 mM NaCl, 10 mM MgCl2, 10% glycerol, 1% Triton-X100 and a cocktail of protease inhibitors (Roche) and centrifuged at 15,000g for 15 min at 4°C. Supernatants were incubated with GST (20 μg/reaction) or GST−LZII (37 μg/reaction) for 15 min at 4°C in the presence of 0.5% BSA. Then, glutathione-Sepharose beads were added for 1 h at 4°C. Beads were washed and bound proteins were analyzed by SDS−PAGE and immunoblotting using anti-ARF6 monoclonal antibody.

### siRNA treatment and lentiviral transduction for protein expression

siRNA transfection was performed using 50 nM siRNA with Lullaby reagent (OZ Biosciences) according to manufacturer’s instructions and analyzed 72 hrs after treatment. The siRNAs used in this study are listed in S2 Table.

For lentivirus production, HEK293T cells were transfected using GeneJuice (Novagen) with a mix of ARF6-T157N expression vector and psPAX2 (AddGene) and pVSV-G (Clonetech) packaging vectors in OPTIMEM (Invitrogen). After 72 hrs, virus-containing supernatant was collected, filtered and used for transduction of a subconfluent monolayer of MDA-MB-231 cells.

### Indirect immunofluorescence and epifluorescence microscopy

MDA-MB-231 cells stably expressing ARF6T15TN were cultured on gelatin-coated coverslips prepared as described [36]. Cells were permeabilized with 0.5% Triton-X100 and 4% PFA in PBS for 90 seconds, fixed in 4% PFA in PBS and stained with indicated antibodies. For β1-integrin staining cells were fixed in 4% PFA in PBS and permeabilized with 0.05% saponin in PBS. For Rac1 staining MDA-MB-231 cells, cultured on gelatin-coated coverslips, were fixed in 4% PFA in PBS and permeabilized with Triton-X100 0.1% in PFA for 4 min before staining. Cells were imaged with the 60X objective of a wide-field microscope DM6000 B/M (Leica Microsystems) equipped with a CCD CoolSnap HQ camera (Roper Scientific) and steered by Metamorph (Molecular Devices Corp., Sunnyvale, CA).

For quantification of Rac1 recruitment at cell edge, a line of 160 pixels was drown perpendicularly to the leading edge in a way that half of the line (about 80^th^ pixel) was at the level of the plasma membrane and fluorescence intensity along the line was measured with the Linescan tool of Metamorph software. Intensity profiles from different cells were then averaged and normalized for the highest value (set to 100).

### Fluorescent gelatin degradation assay

MDA-MB-231 cells and cells stably expressing ARF6T157N were incubated for 5 h on FITC-conjugated cross-linked gelatin (Invitrogen), fixed and stained for cortactin [36]. Cells were imaged with the x63 objective of a wide-field microscope DM6000 B/M (Leica Microsystems) equipped with a CCD CoolSnap HQ camera (Roper Scientific) and steered by Metamorph (Molecular Devices Corp., Sunnyvale, CA). For quantification of degradation, the total area of degraded matrix in one field (black pixels) measured with the Threshold command of MetaMorph was normalized by the total surface of the cell to define a degradation index. The index was then normalized to the one of control siNT-treated cells set to 100.

### Live-cell spinning disk confocal microscopy

MDA-MB-231 cells transiently transfected with DsRed-cortactin and ARF6T15TN-GFP or GFP-Rac1G12V were plated on glass-bottom MatTek dishes coated with cross-linked gelatin and kept in a humidified atmosphere at 37°C and 1% CO2. Formation of cortactin-positive rosettes was monitored by acquiring 3D time-series images by confocal spinning-disk microscopy (1 z-stack/4 or 3 s) using a Nikon Eclipse TE2000-U microscope equipped with a 60x 1.45NA oil immersion objective, a PIFOC Objective stepper, a Yokogawa CSU22 confocal unit and a Roper HQ2 CCD camera steered by Metamorph and a temperature controller. MDA-MB-231 cells stably expressing ARF6T157N and transiently transfected with GFP-cortactin were plated on MatTek dishes layered with a drop of polymerized type I collagen mixed with AlexaFluor 549-conjugated type I collagen (BD) at a final concentration of 2.5 mg/ml. Cells were incubated for 30 min at 37°C in 1% CO2 and then imaged by confocal spinning disk microscopy (2 z-stack/min).

### Total interference reflection fluorescence microscopy (TIRFM)

MDA-MB-231 cells stably expressing ARF6T15TN and transiently transfected with DsRed-cortactin were plated on glass-bottom MatTek dishes coated with cross-linked gelatin for two hours before being imaged. MDA-MB-231 cells were depleted with non-targeting or ARF6 siRNAs and transfected with GFP- or DsRed-cortactin, respectively. The two cell populations (red and green) were mixed and plated on gelatin-coated MatTek dishes for two hours and imaged. Simultaneous dual color TIRFM time-lapse sequences were acquired on a Nikon TE2000 inverted microscope equipped with a ×100 TIRF objective (NA = 1.47), a TIRF arm, an image splitter (DV, Roper Scientific) installed in front of the CCD camera and a temperature controller. GFP and DsRed were excited with 491- and 560 nm-lasers, respectively (100 mW, Roper Scientific), controlled for power by an acousto-optic tunable filter. Fluorescent emissions were selected with bandpass and longpass filters (Chroma) and captured by a QuantEM EMCCD camera (Roper Scientific). The system was driven by Metamorph. Images were acquired with a 1 min interval for several hours. Kymographs were obtained with Metamorph software along a line perpendicular to protruding leading edge. Membrane protrusion speed was calculated by dividing the length of the vertical axis of the kymograph representing the distance of protrusion extension by the time.

For EGF stimulation experiment, MDA-MB-231 cells were transiently transfected with DsRed-cortactin, serum-starved for 16 hours and plated on glass-bottom dishes coated with cross-linked unlabeled gelatin and images were acquired with a 5 sec interval for 1 min before adding EGF (Preprotech), added directly to the medium at 100 ng/mL final concentration.

### EGF stimulation experiments and G-LISA activation assays

MDA-MB-231 cells were serum starved for 12–16 hours and EGF was added at 100 ng/mL final concentration for the indicated times. Cells were transferred on ice and immediately permeabilized with 0.5% Triton-X100 in 4% PFA in PBS for 90 seconds, fixed in 4% PFA in PBS and stained for cortactin. For measuring ARF6 and Rac1 activation levels, we used G-LISA-ARF6 Activation Assay kit (Cytoskeleton Inc, BK133) and G-LISA-Rac1 Activation Assay kit (Cytoskeleton Inc, BK126). MDA-MB-231 cells were treated with the indicated siRNAs for 72 hours, plated on gelatin-coated wells of a 6-well plate, serum starved and EGF-stimulated as described above. EGF stimulation was blocked on ice and cells were immediately lysed with lysis buffer and processed following the manufacturer’s instructions. Briefly the G-LISA kits contain an ARF6- or Rac1-GTP-binding protein linked to the wells of a 96 well plate. Active, GTP-bound ARF6 or Rac1 in cell lysates bind to the wells while the inactive GDP-bound form is removed during washing steps. Active bound ARF6 or Rac1 were detected with ARF6 or Rac1 specific antibodies and the signal is developed with HRP detection reagents following manufacturer’s instructions.

### Statistics

Statistical analyses were performed using Student’s or Mann-Whitney t-test two-way ANOVA and Spearman correlation test using GraphPad Prism (GraphPad Software) as specified in each figure legend with *p* < 0.05 considered significant.

## Results and Discussion

MDA-MB-231 cells stably expressing a fast-cycling mutant of ARF6 (ARF6T157N) were generated by lentiviral transduction. This mutant is characterized by increased spontaneous GDP dissociation and GTP association [34], thus mimicking the effect of hyperactivation of ARF6 due to reported up-regulation of GEP100/BRAG2 ARF6-GEF in highly aggressive breast carcinoma cells [18]. As assessed by pull-down assay using the ARF6-binding domain of JIP3 protein [35], stable expression of ARF6T157N resulted in a ~4-fold increase in GTP-ARF6 levels in MDA-MB-231 cells, while expression of ARF6T27N correlated with almost undetectable GTP-ARF6 levels in agreement with the dominant inhibitory potential of this variant (S1 Fig.).

**Fig 1:**
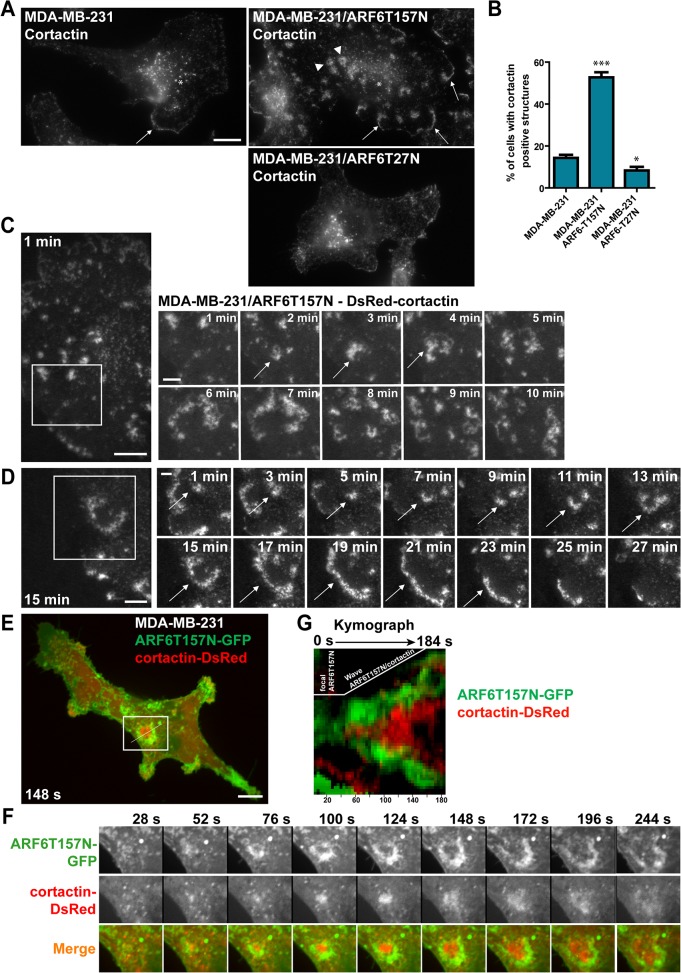
Hyperactivation of ARF6 induces formation of ventral self-expanding F-actin and cortactin-rich rosette-like structures in MDA-MB-231 breast adenocarcinoma cells. **(A)** Immunofluorescence microscopy micrographs of MDA-MB-231 cells (left panel) or MDA-MB-231 cells stably expressing ARF6T157N (upper right panel) or ARF6T27N (lower right panel) stained for cortactin. Arrowheads, ventral cortactin-positive rosettes; arrows, cortactin-enriched lamellipodia; asterisks, endosomal cortactin-rich puncta. Scale bar, 5 μm. **(B)** Percentage of cells displaying cortactin-positive rosettes was scored in the three cell populations. Values are mean ± SEM from three (MDA-MB-231 and MDA-MB-231/ARF6T27N cells) and seven (MDA-MB-231/ARF6T157N cells) independent experiments, scoring 50 cells for each cell population in each experiment. Comparisons were made with a Student’s t-test. ***, P < 0.001, *, P < 0.05 as compared to parental MDA-MB-231 cells. **(C-D)** Still images of TIRFM time-lapse sequences of MDA-MB-231 cells expressing ARF6T157N plated on gelatin. Cells were transiently transfected to express DsRed-cortactin. Scale bars, 10 μm. Galleries correspond to the boxed regions of the still images. Time is in min. Scale bars, 5 μm. **(E)** Still image of a time-lapse sequence of MDA-MB-231 cell expressing ARF6T157N-GFP (green) and cortactin-DsRed (red) plated on gelatin and imaged by confocal spinning disk microscopy. Scale bar, 10 μm. **(F)** The gallery corresponds to the boxed region in E. Time is in seconds. Scale bar, 5 μm. **(G)** Kymograph of ARF6T157N-GFP (green) and cortactin-DsRed (red)-positive rosette. The line used for kymograph analysis is shown in the still image in E.

MDA-MB-231 cells stably expressing ARF6T157N were plated on a 2D substrate of cross-linked gelatin [37] and stained for F-actin and F-actin-binding protein cortactin. Both in ARF6T157N-expressing cells and parental MDA-MB-231 cells, F-actin and cortactin accumulated in peripheral lamellipodia (Fig. 1A, arrows) and in cytoplasmic dot-like structures (Fig. 1A, asterisks) corresponding to F-actin/cortactin double-positive puncta associated with endocytic compartments [38]. ARF6T157N-expressing cells, displayed increased number of cortactin-positive peripheral lamellipodia (Fig. 1A, arrows). In addition, we observed a striking accumulation of F-actin and cortactin in podosome rosette-like structures forming at the ventral cell surface (Fig. 1A and S2 Fig.). Rosette formation occurred in ~50% of the cells as compared to only 14% in parental MDA-MB-231 cells (Fig. 1B). In contrast, expression of dominant inhibitory ARF6T27N led to a 40–50% reduction of cells forming podosome rosettes (Fig. 1AB).

By live-cell imaging by TIRF microscopy (TIRFM) we confirmed that cortactin-positive rosettes occurred ventrally and were highly dynamic, expanding outward as waves and then disassembling in a few minutes (Fig. 1C and S1 Video). When these structures formed in proximity to the cell edge, they often merged with the peripheral plasma membrane (Fig. 1D and S2 Video). This observation, together with the increased number of cortactin-positive peripheral lamellipodia observed in fixed samples (Fig. 1A, arrows) led us to hypothesize an implication for these structures in lamellipodium formation. In agreement with this hypothesis the projected surface area of ARF6T157N-expressing cells was larger (3163 ± 102 μm^2^, scoring 25 cells) than that of parental cells (1654 ± 83 μm^2^, scoring 23 cells) suggesting that activated ARF6-induced cortactin-rich rosettes contribute to cell spreading. c-Src protein Tyrosine-kinase and phosphorylated-tyrosine residues (S2 Fig.) as well as focal adhesion proteins paxillin, vinculin and β1-integrin (S2 Fig.) were associated with cortactin-rich rosettes, suggesting a link with cell adhesion. This assumption was supported by the observation that when MDA-MB-231/ARF6T157N cells were seeded atop of a layer of fibrillar type I collagen, recruitment of cortactin occurred in linear structures forming in association with the underlying collagen fibril and then expanded outwardly as a wave (S2 Fig. and S3 Video). Dynamics of C-terminally GFP-tagged ARF6T157N and cortactin-DsRed were analyzed by dual-color confocal spinning-disk microscopy in MDA-MB-231 cells (Fig. 1E and S4 Video). Focal accumulation of ARF6T157N-GFP occurred at the ventral plasma membrane and grew outward as a self-propagating wave within the plane of the plasma membrane. Live cell imaging and kymograph analysis revealed that a front wave of ARF6T157N preceded a back wave of F-actin/cortactin polymerization (Fig. 1F,G).

**Fig 2:**
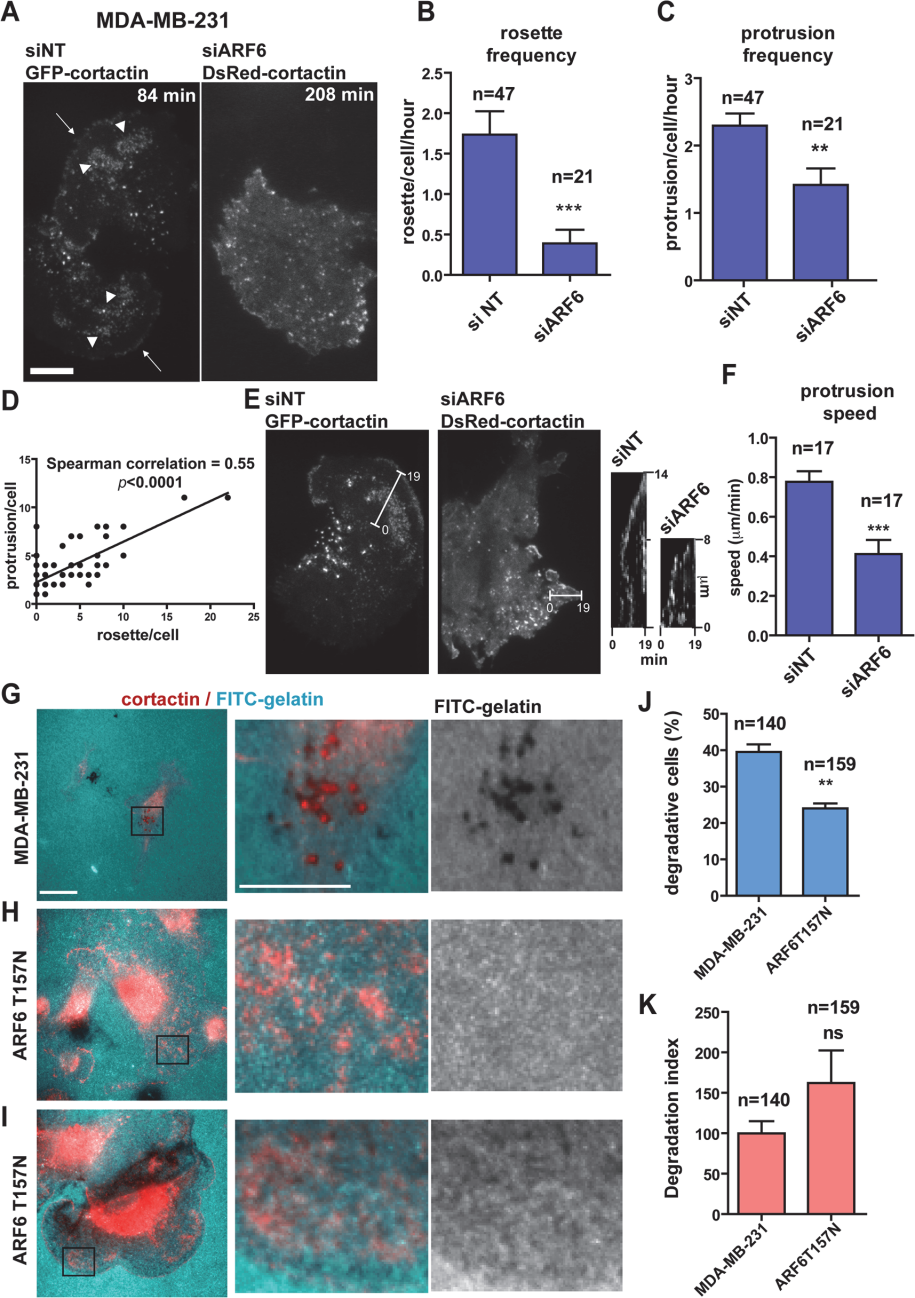
Cortactin-rich rosettes are ARF6-dependent and correlate with membrane protrusion formation. **(A)** Still images of TIRFM time-lapse sequences of MDA-MB-231 cells treated with non-targeting or ARF6 siRNAs and transfected with GFP- or DsRed-cortactin, respectively. Arrowheads, ventral cortactin-positive rosettes; arrows, cortactin-enriched lamellipodia. Scale bars, 10 μm. **(B-C)** Frequency of cortactin-positive rosette **(B)** and membrane protrusion formation **(C)** in the indicated cell populations was calculated by scoring rosettes or protrusions occurring per cell and per hour. Values are mean ± SEM from two independent experiments. *n* represents the number of cells scored for each cell population. Comparisons were made with a Mann-Whitney (two-tailed) t-test. ***, P < 0.001, **, P < 0.01 (compared with siNT-treated cells). **(D)** Correlation between number of rosettes (x-axis) and protrusions (y-axis) scored during time-lapse sequences of control siNT-treated cells (n = 47). Correlation was calculated with a Spearman test. **(E)** Still images of TIRFM movies of MDA-MB-231 cells treated as in A showing extension of membrane protrusions. Kymograph views were obtained from the white lines in the still images between the first and twentieth frame with a 1 min interval between each frame. **(F)** Speed of membrane protrusion extension in the indicated cell populations was calculated by dividing the distance of protrusion (vertical axis in the kymographs) by the time (horizontal axis). Values are mean ± SEM from two independent experiments. *n* represents the number of protrusions scored for each cell population. Comparisons were made with a Mann-Whitney (two-tailed) t-test. ***, P < 0.001 (compared with siNT-treated cells). **(G-I)** Epifluorescence images of MDA-MB-231 cells **(G)** and ARF6T157N-expressing cells **(H-I)** stained for cortactin (red) and plated on FITC-labelled gelatin (cyan). Scale bar 10 μm. Magnification of the boxed regions of the merged images and gelatin channel are shown in the right panels. Scale bar 5 μm. **(J, K)** Percentage of degradative cells **(J)** and degradation index **(K)** calculated by normalizing the degradation area by the cell area in the different cell populations. Values are mean ± SD from two replicate values from one experiment. *n* represents the number of cells scored for each cell population. Comparisons were made with Student’s t-test. Ns, non significant, **, P < 0.01 (compared with MDA-MB-231 cells).

A role for ARF6 in lamellipodia extension was addressed by following the dynamics of cortactin-enriched rosettes in MDA-MB-231 cells by TIRFM (Fig. 2A). In control cells treated with an irrelevant non-targeting siRNA sequence (siNT), ventral rosettes formed in proximity of plasma membrane edge protrusions with a frequency of ~1.7 rosette/hr; silencing of ARF6 drastically decreased rosette formation frequency (Fig. 2AB and S5 Video). Similarly, silencing of ARF6 reduced the frequency of plasma membrane protrusions (Fig. 2C) suggesting a correlation between occurrence of the two structures. Indeed, a positive correlation was observed between the number of rosettes and protrusions forming per cell (Fig. 2D). Finally, kymograph analysis showed that in cells depleted for ARF6, plasma membrane protruded at reduced speed as compared to siNT-treated cells (Fig. 2EF and S6 Video). All together, these data suggest that ARF6-dependent ventral rosettes play a role in membrane protrusions formation and cell motility in MDA-MB-231 breast cancer cells.

The presence of invadopodia-promoting c-Src and phosphorylated-tyrosine residues in podosome-like rosettes (S2 Fig.), together with their morphological similarity with podosomes in Src-transformed cells [39, 40] suggested that ARF6T157N-induced rosettes may represent some type of matrix degradative structures. However, in contrast to *bona fide* cortactin-positive invadopodia forming at the ventral plasma membrane of MDA-MB-231 cells plated on fluorescently labeled gelatin (Fig. 2G), gelatin degradation was not associated with ventral cortactin-positive rosettes in MDA-MB-231/ARF6T157N cells (Fig. 2H). In addition, the percentage of MDA-MB-231/ARF6T157N cells able to degrade gelatin was decreased by 40% as compared to parental MDA-MB-231 cells (Fig. 2J). Of note, a subset of degradative ARF6T157N-expressing cells (~25%) were associated with extensive diffused gelatin degradation (Fig. 2I), contrasting with typical focal invadopodia-mediated degradation in MDA-MB-231 cells (Fig. 2G). Consequently, the degradation index of ARF6T157N-expressing cells increased as compared to parental MDA-MB-231 cells although not significantly (Fig. 2K). Therefore, although we do not exclude the possibility that ventral rosettes may have some degradative capacity, our data rather suggest that because of high intrinsic dynamics, ARF6T157N-induced podosome-like rosettes cannot mediate efficient matrix degradation in contrast to classical long-lasting invadopodia (lifetime > 1 hr, see [41]).

**Fig 3:**
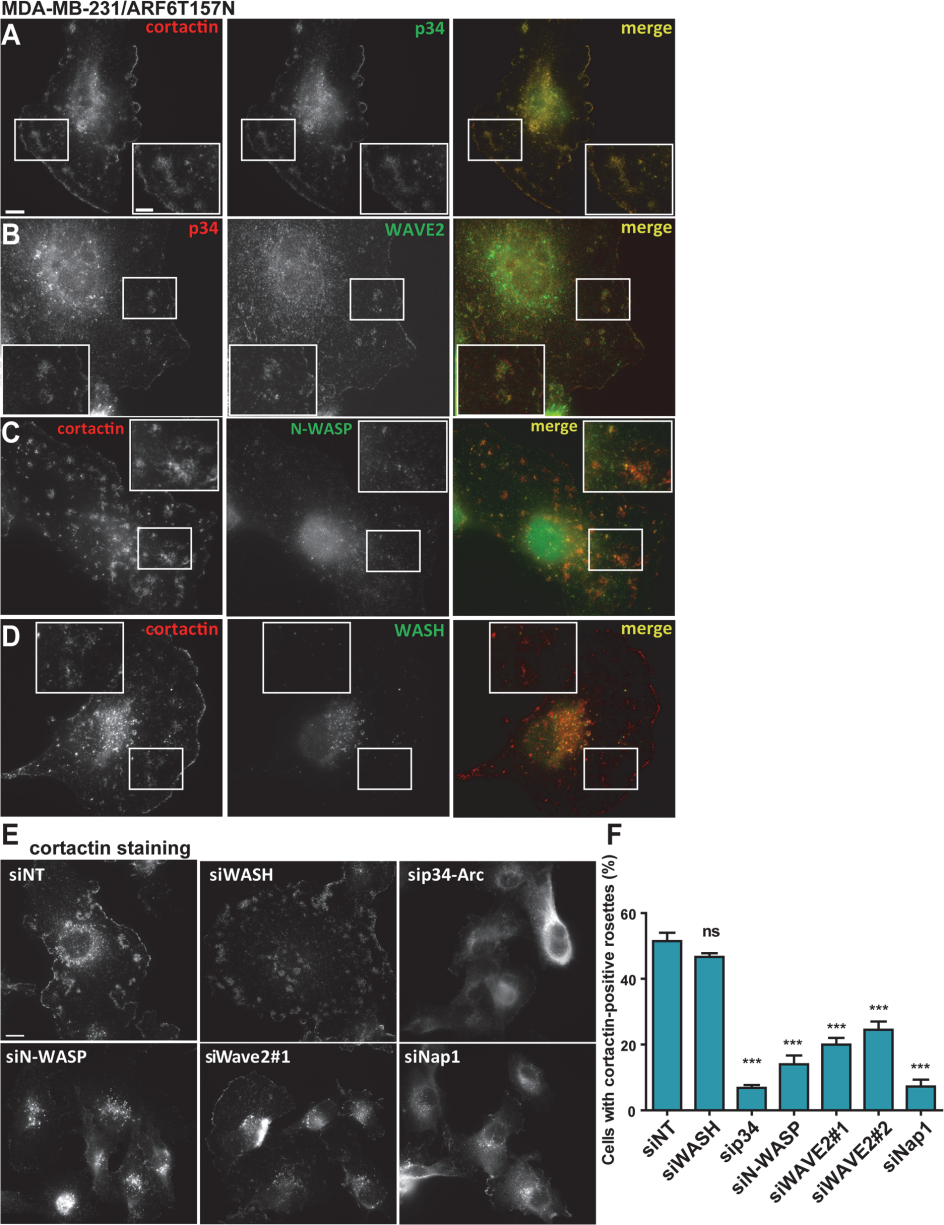
Formation of cortactin-positive ventral rosettes requires the Arp2/3 complex and SCAR/WAVE. **(A-D)** MDA-MB-231 cells stably expressing ARF6T157N plated on cross-linked gelatin were fixed and stained for the indicated proteins. Images were acquired by wide-field microscopy. Scale bar, 10 μm. Insets are higher magnification of the boxed regions. Scale bar 5 μm. **(E)** Cells stably expressing ARF6T157N treated with the indicated siRNAs for 72 hours were plated on cross-linked gelatin, fixed and stained for cortactin. Scale bar, 10 μm. **(F)** The percentage of cells displaying cortactin-positive rosettes was scored. Values are mean ± SEM from at least four independent experiments, scoring about 200 cells for each cell population. Comparisons were made with a Student’s t-test. ns, non significant, ***, P < 0.001 (compared to siNT-treated cells).

We then sought to identify the molecular pathway involved in cortactin-rich rosette formation. We observed that ventral rosettes in MDA-MB-231/ARF6T157N cells were positive for the p34-Arc subunit of the actin-nucleating Arp2/3 complex (Fig. 3A), suggesting that they depend on the branched-dendritic actin machinery for their formation. We investigated the distribution of nucleation promoting factors (NPFs) known to activate the Arp2/3 complex *i*.*e*. WASP family proteins N-WASP, SCAR/WAVE and WASH. WAVE2, a subunit of the SCAR/WAVE complex, showed the strongest co-localization with these structures (Fig. 3B). N-WASP was also present in cortactin-positive rosettes (Fig. 3C). On the contrary, WASH was not detected in ventral rosettes in MDA-MB-231/ARF6T157N cells while it was associated with cortactin-positive endosomal puncta in the central cell region (Fig. 3D).

The contribution of these different proteins to rosette formation was assessed by siRNA-based silencing (S3 Fig.). Silencing of the p34-Arc Arp2/3 complex subunit dramatically decreased the percentage of cells displaying cortactin-rosettes (Fig. 3EF). Knockdown of WAVE2 with two independent siRNAs decreased rosette formation (Fig. 3EF). Pentameric SCAR/WAVE complex exists in several variants and three distinct WAVE subunit isoforms can assemble into different complexes [42]. Knockdown of Nap1, a common subunit to all SCAR/WAVE complexes, resulted in drastic inhibition of cortactin-positive rosettes formation (Fig. 3EF). Given the prominent role of SCAR/WAVE in lamellipodia regulation and cell motility downstream of Rac [43–46], these data suggested a role for ARF6 as an upstream regulator of the SCAR/WAVE complex and therefore of lamellipodia formation in breast cancer cells. In agreement with localization data, WASH knockdown did not cause any significant effect, while silencing of N-WASP decreased the percentage of cells displaying cortactin-positive rosettes from 50% in control cells to 20% suggesting a possible implication of N-WASP in ventral rosette formation downstream of activated ARF6 (Fig. 3EF). These findings are compatible with the reported localization of N-WASP in lamellipodia in carcinoma cells [5] and with a possible role for N-WASP in lamellipodia regulation [7]. Finally, inhibition of rosette formation in cells silenced for the Arp2/3 complex or SCAR/WAVE and N-WASP NPFs correlated with a strong decrease in cell area (see Fig. 3E), in agreement with a role for ARF6-mediated rosette formation in lamellipodia extension and cell spreading and adhesion.

**Fig 4:**
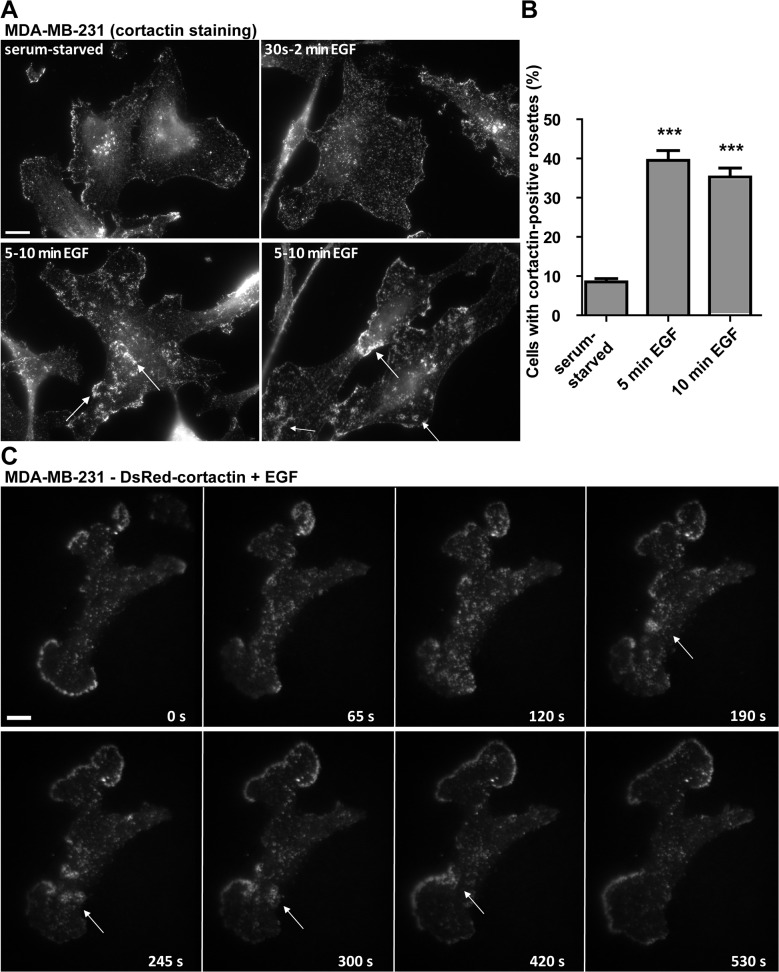
EGF stimulation triggers the formation of ventral F-actin structures in MDA-MB-231 cells. **(A)** MDA-MB-231 cells were plated on cross-linked gelatin, serum starved over-night and stimulated with EGF for 30’ sec up to 10 min as indicated. Then cells were fixed and stained for cortactin and images were acquired by wide-field microscopy. Arrows point to nascent cortactin-positive rosettes. Scale bar, 10 μm. **(B)** MDA-MB-231 cells were treated as in **A** and the percentage of cells displaying cortactin-positive rosettes was scored. Values are mean ± SEM from duplicate samples in two independent experiments scoring 100 cells for each cell population in each experiment. Comparisons were made with a Student’s t-test. ***, P < 0.001 (compared with serum-starved MDA-MB-231 cells). **(C)** Gallery from a time-lapse sequence of MDA-MB-231 cells expressing DsRed-cortactin after EGF-treatment. Arrows point to nascent cortactin-positive rosette. Time is in sec. Scale bar, 10 μm.

Since EGF-R signaling has been implicated in the formation of migratory protrusions, such as lamellipodia and CDRs [8, 13, 27] and in ARF6 activation [18], we investigated whether EGF treatment could result in rosette formation in serum-starved MDA-MB-231 cells. Using wide-field fluorescence microscopy, we observed a rearrangement of cortactin distribution already 30 sec-2 min after EGF stimulation and formation of cortactin-positive rosettes was visible in up to 40% of the cells after 5–10 min (Fig. 4AB). Using TIRFM, we confirmed that EGF-induced rosettes occurred at the ventral cell surface (Fig. 4C and S7 Video) with similar morphology and dynamics as compared to ARF6T157N-induced structures.

**Fig 5:**
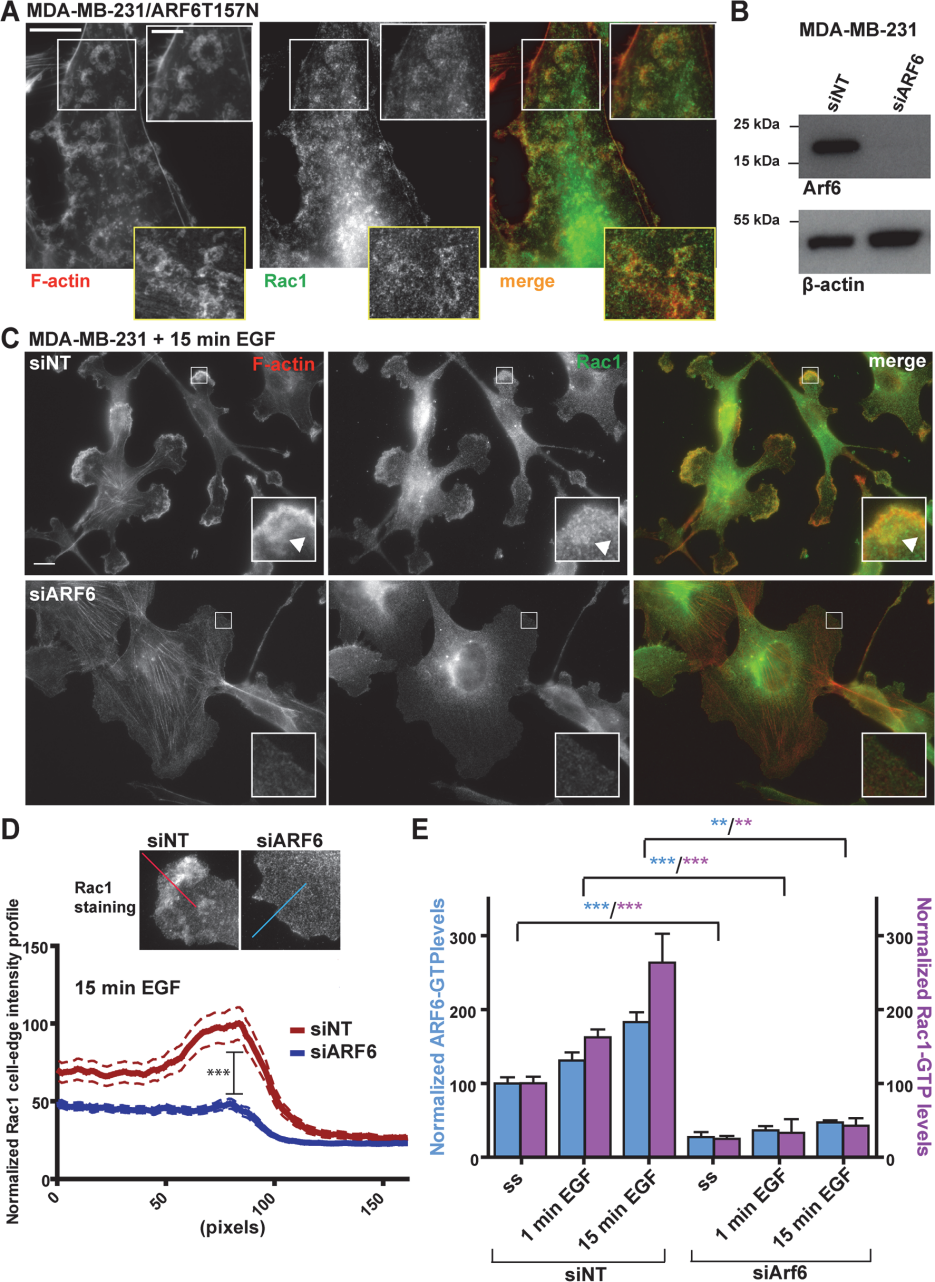
Rac1 localizes in ventral actin-rich rosettes and ARF6 is required for Rac1 activation and recruitment. **(A)** MDA-MB-231 cells stably expressing ARF6T157N were plated on cross-linked gelatin, fixed and stained for F-actin and Rac1. Upper insets are higher magnifications of boxed regions. Lower insets show rosettes from another cell. Scale bars, 10 μm and 5 μm (insets). **(B)** ARF6 immunoblotting analysis of MDA-MB-231 cell lysates after indicated siRNA treatments. Anti-β-actin was used as loading control. **(C)** MDA-MB-231 cells were treated with the indicated siRNAs for 72 hours, plated on gelatin and then serum-starved for 12–16 hrs. After 15 min treatment with EGF, cells were fixed and stained for F-actin and Rac1. Insets are higher magnification of boxed regions. Scale bars, 10 μm. Arrowheads, peripheral F-actin-rich rosette. **(D)** Quantification of Rac1 fluorescence intensity profile was done along a 160-pixel line drawn perpendicularly to the cell edge as shown in insets. Intensity profiles along the line from at least 50 cells per condition from two independent experiments were averaged and normalized to the highest fluorescence intensity value in the siNT-treated cells. Comparison between the two mean peak values (corresponding to the cell edge) was made with a two-way ANOVA test. ***, P < 0.001 (compared to siNT-treated cells). **(E)** MDA-MB-231 cells were treated with the indicated siRNAs for 72 hrs, plated on gelatin, serum-starved and treated with EGF for 1 or 15 min. Levels of GTP-bound ARF6 (blue bars) and Rac1 (purple bars) in the different conditions were measured with G-LISA assay. Values are normalized mean ± SEM from replicate samples from two independent experiments. Comparisons were made with a Student’s t-test. **, P < 0.01, ***, P < 0.001 (compared with siNT-treated cells).

These data suggested that EGF-R triggers the formation of cortactin-rich ventral rosettes through activation of ARF6 that could be mimicked by expression of the fast-cycling ARF6T157N variant. Thus, we hypothesized that in breast cancer cells, ARF6 could be activated downstream of EGF-R signaling and activates SCAR/WAVE and Arp2/3 complexes with a potential regulatory role in leading edge extension and adhesion to the ECM. As Rac1 is the most prominent activator of the SCAR/WAVE complex and because ARF6 has been reported to regulate Rac1 activation, we looked at a possible regulation of Rac1 by ARF6 in MDA-MB-231 cells. We observed that in MDA-MB-231 cells stably expressing ARF6T157N, Rac1 colocalized with ventral cortactin-positive rosettes (Fig. 5A) and levels of active GTP-bound Rac1 increased by 50% as compared to control cells (S4 Fig.). Moreover expression of constitutively active GFP-tagged form of Rac1 (Rac1GV12) in MDA-MB-231 triggered formation of ventral cortactin-positive rosettes (S4 Fig. and S8 Video) similar to those induced upon expression of ARF6T157N or EGF treatment. All together, these observations suggested that Rac1 activation is required for cortactin-positive rosette formation downstream of ARF6. Along this line, we investigated a potential role of ARF6 in spatial polarization of Rac1 distribution by silencing ARF6 in MDA-MB-231 cells (Fig. 5B). By immunofluorescence staining we observed that EGF stimulation of MDA-MB-231 cells for 15 minutes induced increased lamellipodial recruitment of Rac1 and F-actin (Fig. 5C, upper panels). F-actin- and Rac1-enriched rosettes were also visible although they were mainly peripheral after 15 minutes of EGF stimulation (Fig. 5C, arrowheads). In contrast, silencing of ARF6 resulted in a 50% reduction of lamellipodial recruitment of Rac1 and inhibition of lamellipodia formation (Fig. 5C,D). Based on these data, we propose that ARF6 regulates localized targeting and activation of Rac1 to the leading edge of breast cancer cells where in turn, activated Rac1 triggers lamellipodial actin polymerization. We used ARF6 and Rac1 G-LISA activation assays to monitor the activation level of both GTP-binding proteins under EGF stimulation of serum-starved MDA-MB-231 cells. Both ARF6 (Fig. 5E, blue bars) and Rac1 activation levels (Fig. 5E, purple bars) increased by 1 min and further by 15 min EGF treatment. Moreover, silencing of ARF6 decreased by six-fold the level of GTP:Rac1 both in non-stimulated condition and in response to EGF stimulation and abolished EGF-induced Rac1 activation (Fig. 5E). All together, these findings strongly suggest that ARF6 is required for Rac1 activation and localization.

Although it is not clear how ARF6 controls Rac1 activation, our findings indicate that in breast cancer cells, ARF6 regulates Rac1 association to the leading edge, possibly involving a Rac1-GEF and that in absence of ARF6, EGF-mediated localization and activation of Rac1 cannot take place. These data are in agreement with a previous study in HeLa cells showing that Rac1 and its GEF, TIAM1 are internalized into early endosomes and activated Rac1 is recycled to dynamic regions of the plasma membrane in response to hepatocyte growth factor in a ARF6-dependent manner leading to the formation of CDRs [27]. One possibility is that cortactin ventral structures we described could play a role similar to CDRs in actin remodeling for lamellipodia formation and ARF6 could be involved in directed cell motility by controlling the formation of these dynamic actin-based structures depending on specific growth factor receptors through localized activation of Rac1 at the ventral or dorsal plasma membrane.

Recent reports have also described ventral F-actin-based waves in several cell types including Dictyostelium discoideum [47]. In neutrophils, Nap1-positive actin propagating waves are thought to play a role in spatial organization and protrusion of the leading edge during cell motility [48]. Fibroblast and human osteosarcoma cells also exhibit ventral F-actin waves positive for Arp2/3 complex, β1-integrin, paxillin, vinculin and other adhesive proteins such as talin and zyxin that propagate as spots and wave-fronts along the ventral plasma membrane and are thought to play a role in coupling ventral actin polymerization and cycles of integrin adhesion/de-adhesion to the ECM [49]. The present study documents the existence of actin dynamic waves in highly aggressive breast cancer cells and points to ARF6 as a master regulator of wave-like structures. A recent report also demonstrated that expression of active mutant forms of ARF1 or ARF6 triggers formation of ventral F-actin waves in epithelial HeLa and Beas-2b cell lines [50]. Induction of these structures was blocked by PKC and c-Src inhibitors and required PI(4,5)P_2_ [50]. Our observations extend these data by showing that ARF6-induced F-actin ventral rosettes are coupled with plasma membrane protrusions and lamellipodia extension in response to EGF with potential contribution to breast cancer cell motility and matrix remodeling, two essential arms of the metastatic program. In addition, we identified the SCAR/WAVE complex known to control lamellipodia formation, as an essential component of ARF6-mediated rosette formation downstream of Rac1 localization and activation. Along this line, a cascade linking ARF6 and ARF1 in the activation of the SCAR/WAVE complex has been recently reported [51].

## Conclusion

Our study provides further evidence for the known activation and plasma membrane localization of Rac1 by ARF6 [23–27] in breast cancers, suggesting a potential contribution for this pathway during the invasive process. Several questions remain unsolved such as how actin wave can propagate in response to wave of ARF6 activity on the ventral plasma membrane, and whether EGF-R signaling is required only for initiation of ventral ARF6/actin waves or if it must be sustained for wave-front propagation. Future studies will be required to decipher the underlying mechanism and function of ARF6-dependent actin waves and their importance for breast cancer cell migration.

## Supporting Information

S1 Fig Stable expression of ARF6T157N results in increased GTP-ARF6 levels in MDA-MB-231 cells. **(A-B)** MDA-MB-231 cells were stably transduced with lentiviral vectors expressing the indicated ARF6 variants. Cells lysates were incubated with GST alone or GST fused with the ARF6-binding domain (LZII, leucine zipper II domain) of human JIP3 (aa 371–507), which interacts specifically with GTP-bound ARF6. Immunoblotting analysis of ARF6 in the bound (GTP:ARF6) and input fractions. α-tubulin was used as a loading control. **(B)** Densitometric quantification of ARF6 bands in panel A. Values represent GTP:ARF6 levels after normalization to total ARF6 and α-tubulin levels.
(TIF)

S2 Fig Ventral cortactin-positive rosettes are positive for invadopodia markers and ECM adhesion proteins. **(A-C)** MDA-MB-231 cells stably expressing ARF6T157N were plated on cross-linked gelatin, fixed and stained for the indicated markers. Images were acquired with epifluorescence microscopy. Insets are magnification of the boxed regions. Scale bars, 10 μm and 5 μm (insets). **(D)** Still image of a confocal spinning-disk microscopy time-lapse sequence of MDA-MB-231 cells stably expressing ARF6T157N plated on a layer of Alexa-546-conjugated type I collagen fibers (red). Cells were transiently transfected with GFP-cortactin (green). Scale bar, 10 μm. The gallery corresponds to the boxed region in the still image and show a cortactin–positive rosette (arrows) forming in association with a collagen I fiber and propagating as a wave. Time is in min. Scale bar, 5 μm.
(TIF)

S3 Fig Immunoblotting analysis of siRNA-treated cells. **(A-E)** Immunoblotting analysis of lysates of MDA-MB-231 cells stably expressing ARF6T157N treated with indicated siRNAs for 72 hrs. Antibodies are indicated on the right. Immunoblotting analysis with anti-α tubulin and anti β1-integrin was used as loading control. Asterisk in **A** indicates WASH-specific band. **(A'-E')** Densitometric quantification of bands in panels A-E. Values represent mean ± SEM of density levels of each protein normalized for β1-integrin (A'-D') or α-tubulin (E') density values from four (A' and C') and three (B', D' and E’) independent experiments. Comparisons were made with a Student’s t-test. ***, P < 0.001, **, P < 0.01 *, P < 0.05 (compared to siNT-treated cells).
(TIF)

S4 Fig Induction of cortactin-positive rosette by constitutively activated Rac1. **(A)** GTP:Rac1 levels were compared in MDA-MB-231 cells vs. cells stably expressing ARF6T157N. Values are normalized mean ± SEM from replicate samples. Comparison was made with a Student’s t-test. *, P < 0.05 (compared to MDA-MB-231 cells). **(B-C)** Still image (B) and gallery (panel C, corresponding to the boxed region in B) of a time-lapse sequence of a MDA-MB-231 cell transiently expressing Rac1G12V-GFP and cortactin-DsRed plated on cross-linked gelatin and imaged with confocal spinning disk microscopy. Scale bar, 10 μm. The gallery corresponds to the boxed region of the still image and shows formation of cortactin-positive rosettes (red) associated with Rac1G12V-GFP (green). Time is in seconds. Scale bar, 5 μm.
(TIF)

S1 Table Antibodies used in this study. This table provides a list of monoclonal and polyclonal antibodies used in this study, their source and specific use.
(DOCX)

S2 Table siRNAs used in this study. This table provides a list of siRNAs used in this study, their sequence and source.
(DOCX)

S1 Video Dynamics of ARF6T157N-induced cortactin-positive ventral rosettes. MDA-MB-231 cells stably expressing ARF6T157N and transiently transfected with DsRed-cortactin were plated on unlabeled cross-linked gelatin and imaged by TIRFM (Nikon TE2000 inverted). Images were acquired every minute. Scale bar 10 μm.
(MOV)

S2 Video Dynamics of ARF6T157N-induced cortactin-positive rosette located close to the cell edge. MDA-MB-231 cells stably expressing ARF6T157N and transiently transfected with DsRed-cortactin were plated on cross-linked unlabeled gelatin and imaged by TIRFM (Nikon TE2000 inverted). Images were acquired every minute. Scale bar 10 μm.
(MOV)

S3 Video ARF6T157N-induced cortactin-positive ventral rosette forming in association with a type I collagen fibril. MDA-MB-231 cells stably expressing ARF6T157N and transiently transfected with cortactin-GFP were plated on a layer of Alexa Fluor 549–conjugated collagen I fibrils (red) for 30 min and imaged by confocal spinning disk microscopy (inverted, Nikon Eclipse TE2000-U). Images were acquired every 30 sec. Scale bar 10 μm. The inset is a magnification of the boxed region corresponding to the gallery in S2D Fig.
(MOV)

S4 Video Dynamics of ARF6T157N and cortactin in ventral rosette. MDA-MB-231 cells transiently transfected with DsRed-cortactin (red) and ARF6T157N-GFP (green) were plated on cross-linked gelatin and imaged by confocal spinning disk microscopy (inverted, Nikon Eclipse TE2000-U). Images were acquired every 4 seconds. Scale bar 10 μm. The inset is a magnification of the boxed region and corresponds to the gallery shown in Fig. 1F.
(MOV)

S5 Video Dynamics of cortactin-positive ventral rosettes and plasma membrane protrusions in presence or absence of ARF6 in MDA-MB-231 cells. MDA-MB-231 cells treated with non-targeting (left) or ARF6 (right) siRNAs and transfected with GFP- or DsRed-cortactin, respectively, were mixed, plated on unlabeled cross-linked gelatin and imaged by dual-color TIRFM (Nikon TE2000 inverted). Images were acquired every minute. Scale bar 10 μm.
(MOV)

S6 Video Dynamics and speed of plasma membrane protrusions extension in presence or absence of ARF6 in MDA-MB-231 cells. MDA-MB-231 cells treated with non-targeting (left) or ARF6 (right) siRNAs and transfected with GFP- or DsRed-cortactin, respectively, were mixed, plated on unlabeled cross-linked gelatin and imaged by dual-color TIRFM (Nikon TE2000 inverted). Images were acquired every minute. Scale bar 10 μm.
(MOV)

S7 Video EGF stimulation triggers the formation of ventral actin structures in MDA-MB-231 cells. Serum-starved MDA-MB-231 cells transiently transfected with DsRed-cortactin were plated on cross-linked gelatin and imaged by TIRFM (Nikon TE2000 inverted). Images were acquired every 5 sec. EGF was added directly in the medium after 1 min of imaging. Scale bar 10 μm.
(MOV)

S8 Video Induction of cortactin-positive ventral rosettes by Rac1G12V. MDA-MB-231 cells transiently transfected with DsRed-cortactin (red) and Rac1G12V-GFP (green) were plated on cross-linked unlabeled gelatin and imaged by confocal spinning disk microscopy (inverted, Nikon Eclipse TE2000-U). Images were acquired every 3 seconds. Scale bar 10 μm. The inset is a magnification of the boxed region and corresponds to the gallery shown in S4C Fig.
(MOV)
