# Supplementary figures and images for: ARF6 Promotes the Formation of Rac1 and WAVE-Dependent Ventral F-Actin Rosettes in Breast Cancer Cells in Response to Epidermal Growth Factor
Source: PLoS One. 2015 Mar 23;10(3):e0121747. doi: 10.1371/journal.pone.0121747 (PMC4370635; doi:10.1371/journal.pone.0121747)

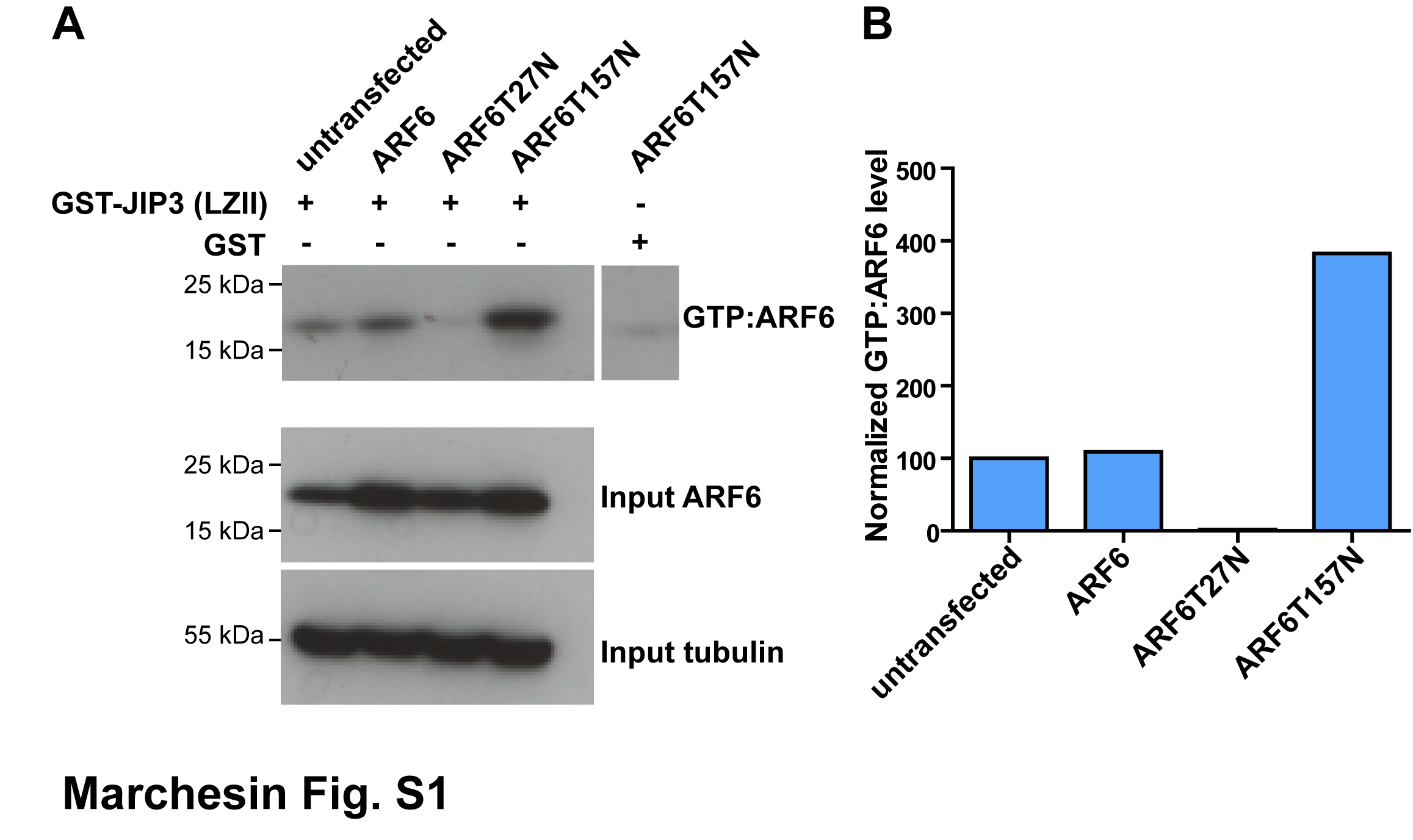

Supplement: S1 Fig — (A-B) MDA-MB-231 cells were stably transduced with lentiviral vectors expressing the indicated ARF6 variants. Cells lysates were incubated with GST alone or GST fused with the ARF6-binding domain (LZII, leucine zipper II domain) of human JIP3 (aa 371–507), which interacts specifically with GTP-bound ARF6. Immunoblotting analysis of ARF6 in the bound (GTP:ARF6) and input fractions. α-tubulin was used as a loading control. (B) Densitometric quantification of ARF6 bands in panel A. Values represent GTP:ARF6 levels after normalization to total ARF6 and α-tubulin levels. (TIF) [file pone.0121747.s001.tif]

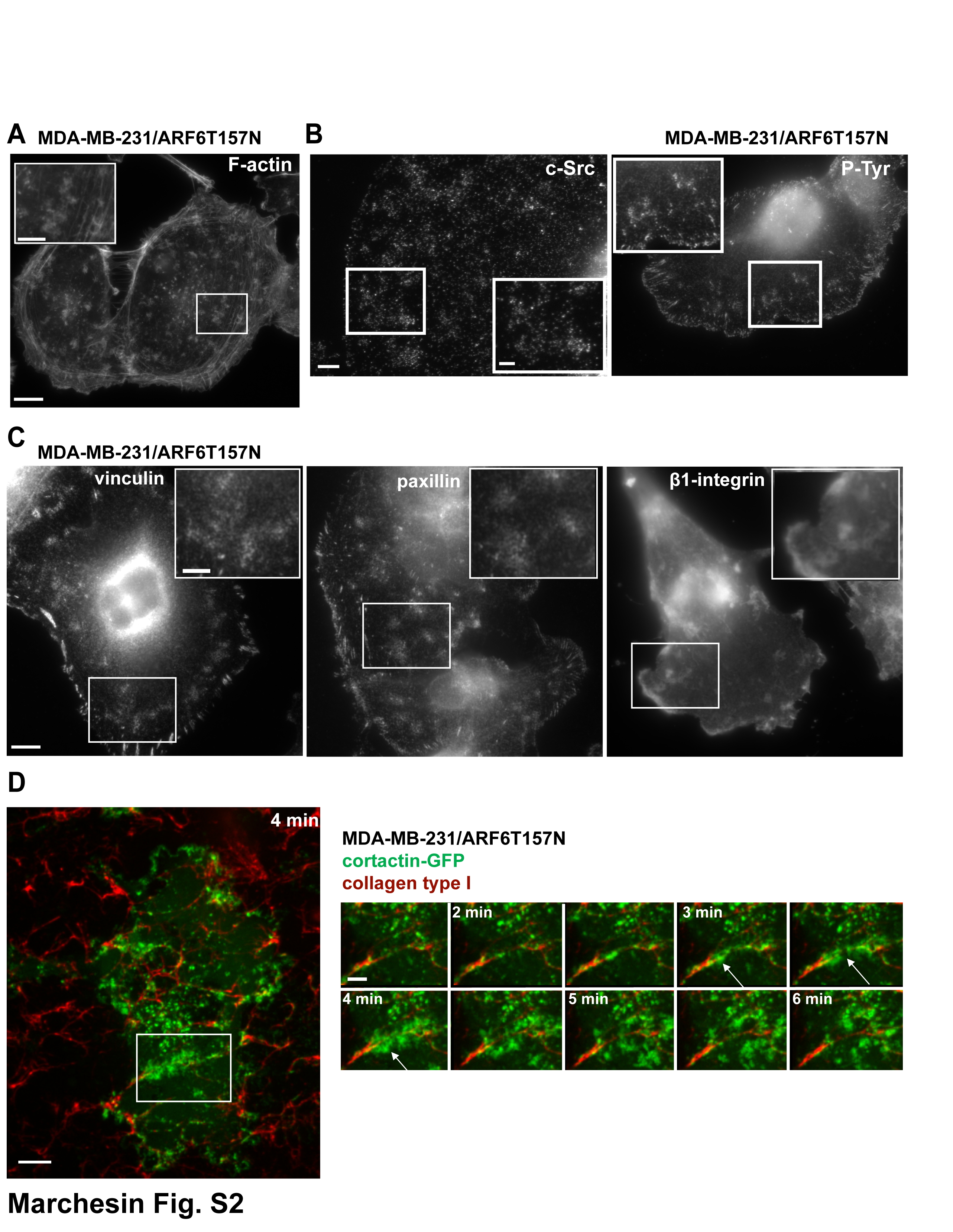

Supplement: S2 Fig — (A-C) MDA-MB-231 cells stably expressing ARF6T157N were plated on cross-linked gelatin, fixed and stained for the indicated markers. Images were acquired with epifluorescence microscopy. Insets are magnification of the boxed regions. Scale bars, 10 μm and 5 μm (insets). (D) Still image of a confocal spinning-disk microscopy time-lapse sequence of MDA-MB-231 cells stably expressing ARF6T157N plated on a layer of Alexa-546-conjugated type I collagen fibers (red). Cells were transiently transfected with GFP-cortactin (green). Scale bar, 10 μm. The gallery corresponds to the boxed region in the still image and show a cortactin–positive rosette (arrows) forming in association with a collagen I fiber and propagating as a wave. Time is in min. Scale bar, 5 μm. (TIF) [file pone.0121747.s002.tif]

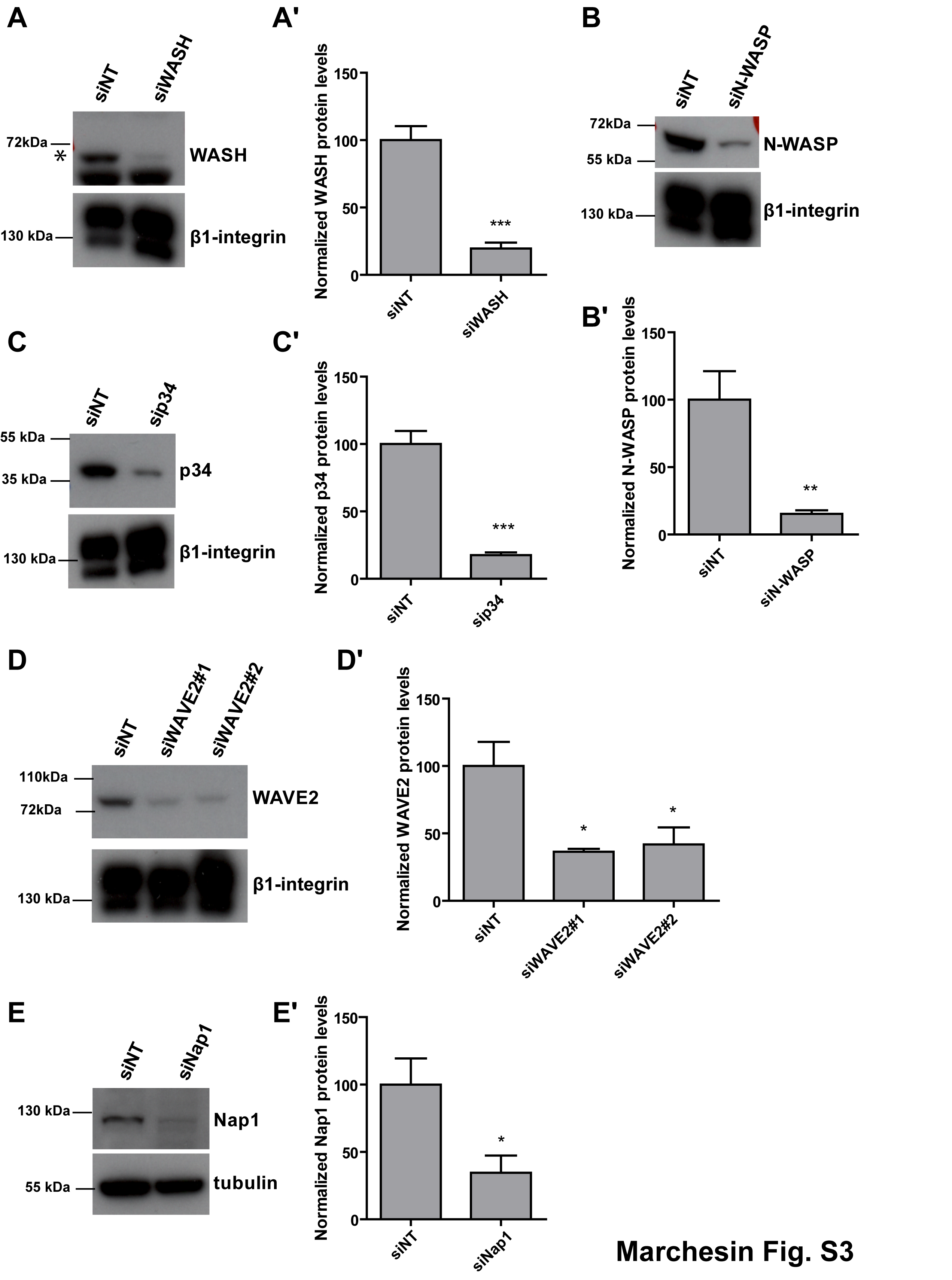

Supplement: S3 Fig — (A-E) Immunoblotting analysis of lysates of MDA-MB-231 cells stably expressing ARF6T157N treated with indicated siRNAs for 72 hrs. Antibodies are indicated on the right. Immunoblotting analysis with anti-α tubulin and anti β1-integrin was used as loading control. Asterisk in A indicates WASH-specific band. (A'-E') Densitometric quantification of bands in panels A-E. Values represent mean ± SEM of density levels of each protein normalized for β1-integrin (A'-D') or α-tubulin (E') density values from four (A' and C') and three (B', D' and E’) independent experiments. Comparisons were made with a Student’s t-test. ***, P < 0.001, **, P < 0.01 *, P < 0.05 (compared to siNT-treated cells). (TIF) [file pone.0121747.s003.tif]

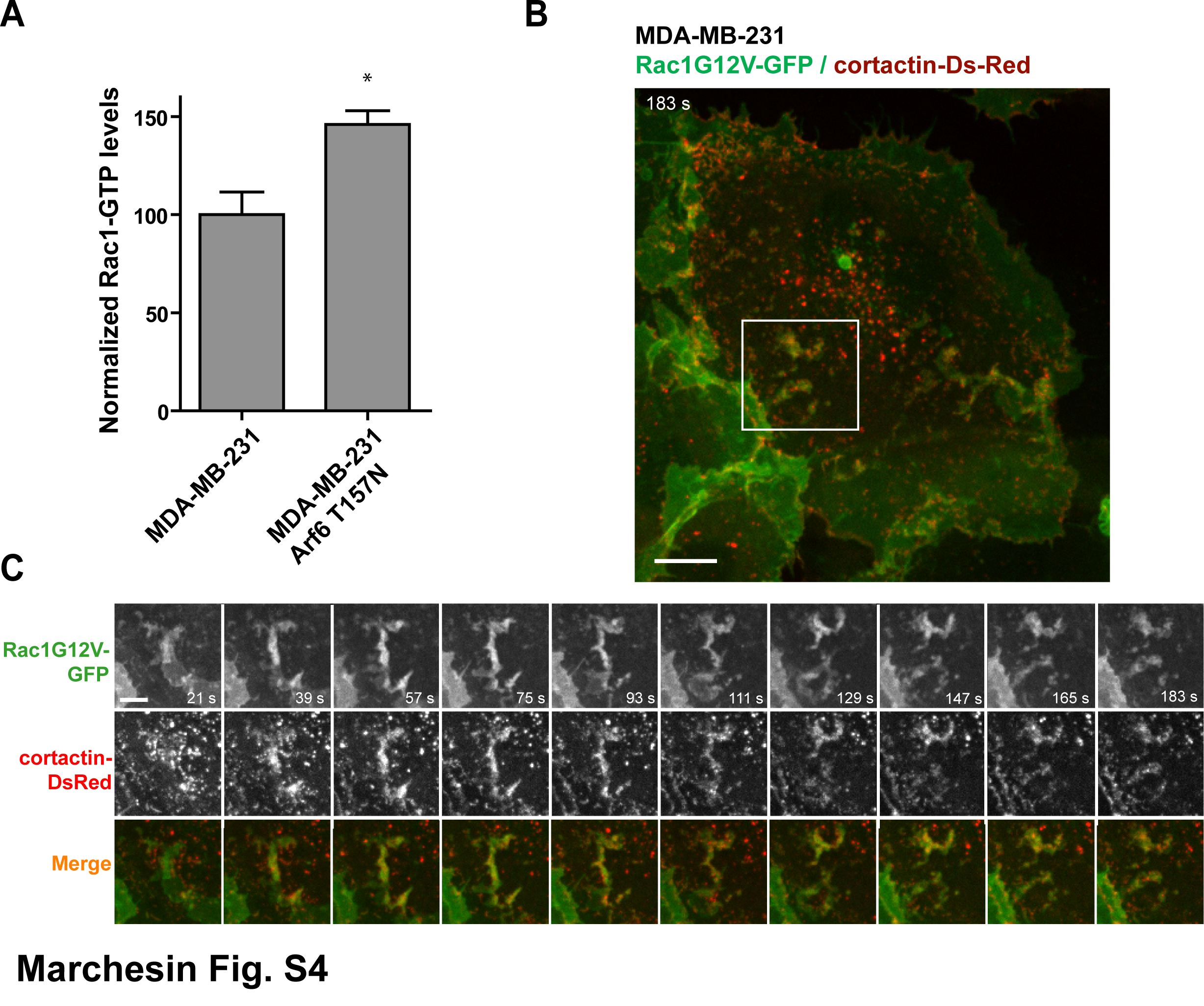

Supplement: S4 Fig — (A) GTP:Rac1 levels were compared in MDA-MB-231 cells vs. cells stably expressing ARF6T157N. Values are normalized mean ± SEM from replicate samples. Comparison was made with a Student’s t-test. *, P < 0.05 (compared to MDA-MB-231 cells). (B-C) Still image (B) and gallery (panel C, corresponding to the boxed region in B) of a time-lapse sequence of a MDA-MB-231 cell transiently expressing Rac1G12V-GFP and cortactin-DsRed plated on cross-linked gelatin and imaged with confocal spinning disk microscopy. Scale bar, 10 μm. The gallery corresponds to the boxed region of the still image and shows formation of cortactin-positive rosettes (red) associated with Rac1G12V-GFP (green). Time is in seconds. Scale bar, 5 μm. (TIF) [file pone.0121747.s004.tif]
