# Supplementary material for: ARF6 Promotes the Formation of Rac1 and WAVE-Dependent Ventral F-Actin Rosettes in Breast Cancer Cells in Response to Epidermal Growth Factor
Source: PLoS One. 2015 Mar 23;10(3):e0121747. doi: 10.1371/journal.pone.0121747 (PMC4370635; doi:10.1371/journal.pone.0121747)
Supplement: S1 Table — This table provides a list of monoclonal and polyclonal antibodies used in this study, their source and specific use. (DOCX) [file pone.0121747.s005.docx]

|  |  |  |  |
| --- | --- | --- | --- |
| Antigen | **Type (Species)** | **Source** | **Use** |
| Cortactin | Monoclonal (Mouse) | Millipore | IF |
| Alexa Fluor–conjugated phalloidin |  | Invitrogen | IF |
| SRC | Polyclonal (Rabbit) | Upstate Biotechnology | IF |
| P-tyrosines | Monoclonal (Mouse) | Upstate Biotechnology | IF |
| paxillin | Monoclonal (Mouse) | Transduction laboratories | IF |
| vinculin | Monoclonal (Mouse) | M.Glukhova (Institut Curie, Paris, France) | IF |
| β-actin | Monoclonal (Mouse) | Sigma | WB |
| β1-integrin | Monoclonal (Mouse) | Beckman Coulter | IF |
| β1-integrin | Polyclonal (Rabbit) | C. Albiges-Rizo, Institut Albert Bonniot, Grenoble, France | WB |
| WAVE2 | Monoclonal (Mouse) | G.Scita (IFOM,Milan, Italy) | IF |
| WAVE2 | Polyclonal (Rabbit) | A. Gautreau (CNRS, Gif-sur-Yvette, France) | WB |
| p34-Arc (ARPC2) | Polyclonal (Rabbit) | Millipore | IF/WB |
| WASH | Polyclonal (Rabbit) | Derivery et al., 2009 | IF/WB |
| N-WASP | Polyclonal (Rabbit) | Cell Signaling Technology | IF/WB |
| Rac1 | Monoclonal (Mouse) | BD Transduction Laboratories | IF |
| α-tubulin | Monoclonal (Mouse) | Sigma | WB |
| Nap1 | Polyclonal (Rabbit) | Upstate Biotechnology | WB |
| Secondary antibodies |  | Jackson ImmunoResearch Laboratories, Inc | IF |
| HRP-conjugated anti-rabbit IgG |  | Sigma | WB |
| HRP-conjugated anti-mouse IgG |  | Jackson ImmunoResearch Laboratories, Inc | WB |
