# Supplementary material for: ARF6 Promotes the Formation of Rac1 and WAVE-Dependent Ventral F-Actin Rosettes in Breast Cancer Cells in Response to Epidermal Growth Factor
Source: PLoS One. 2015 Mar 23;10(3):e0121747. doi: 10.1371/journal.pone.0121747 (PMC4370635; doi:10.1371/journal.pone.0121747)
Supplement: S2 Table — This table provides a list of siRNAs used in this study, their sequence and source. (DOCX) [file pone.0121747.s006.docx]

| siRNA |  |  |
| --- | --- | --- |
| Gene | **Sequence (Sens)** | **Company** |
| ARF6 | 5'-CGGCAUUACUACACUGGGA-3' | Thermo Fisher Scientific |
| WASH | 5'- UGUCGGAUCUCUUCAACAA-3' | Thermo Fisher Scientific |
| NCKAP1 (Nap1) | 5'-GGUCGUAGCUCUUUCUUCA-3',  5'-GGAGAAUGUUGAUGUGUUA-3', 5'-GCAGACGACUUUAUAGAUA-3', 5'-CAUCCUAUCUUAUCGACAA-3' | Thermo Fisher Scientific |
| ARPC2 (p34) | 5'-GUACGGGAGUUUCUUGGUA-3' | Thermo Fisher Scientific |
| WASF2 (WAVE2#1) | 5'- GGGCAGAGCUUUCUCAGUU-3' | Thermo Fisher Scientific |
| WASF2 (WAVE2#2) | 5'-GGAUUUGGGUCUCCAGGGA-3’ | Thermo Fisher Scientific |
| (WASL) N-WASP | 5’-CAGCAGAUCGGAACUGUAU-3’  5’-UAGAGAGGGUGCUCAGCUA-3’  5’-GGUGUUGCUUGUCUUGUUA-3’  5’-CCAGAAAUCACAACAAAUA-3’ | Thermo Fisher Scientific |
| Non targeting |  | Thermo Fisher Scientific |
